# Interaction of *Plasmodium yoelii* tryptophan-rich antigen 7 with CD71 on macrophage membrane regulates host inflammatory response

**DOI:** 10.1016/j.isci.2026.115468

**Published:** 2026-03-25

**Authors:** Yifan Sun, Zhe Chen, Chenyan Du, Yao Lei, Jian Li, Hangye Zhang, Xuan Huang, Bo Wang, Shenghuan Zuo, Zhiyue Lv, Jianping Cao, Su Han, Yang Cheng

**Affiliations:** 1 Department of Laboratory Medicine, Affiliated Hospital of Jiangnan University, Wuxi, Jiangsu, China; 2 Laboratory of Pathogen Infection and Immunity, Department of Public Health and Preventive Medicine, Wuxi School of Medicine, Jiangnan University, Wuxi, Jiangsu, China; 3 School of Food Science and Technology, Jiangnan University, Wuxi, Jiangsu, China; 4 Department of Microbiology Laboratory, Chengdu Center for Disease Control and Prevention, Chengdu, Sichuan, China; 5 State Key Laboratory of Cellular Stress Biology, School of Life Sciences, Faculty of Medicine and Life Sciences, Xiamen University, Xiamen, Fujian, China; 6 Department of Clinical Laboratory, the First Affiliated Hospital of Anhui Medical University, Hefei, Anhui, China; 7 Cancer Research Center Nantong, Affiliated Tumor Hospital of Nantong University, Nantong, Jiangsu, China; 8 Zhongshan School of Medicine, Sun Yat-sen University, Guangzhou, Guangdong, China; 9 National Institute of Parasitic Diseases, Chinese Center for Disease Control and Prevention (Chinese Center for Tropical Diseases Research), NHC Key Laboratory of Parasite and Vector Biology, WHO Collaborating Centre for Tropical Diseases, National Center for International Research on Tropical Diseases, Shanghai, China; 10 Institute for Prevention and Control of Tropical Diseases and Chronic Diseases, Hainan Provincial Center for Disease Control and Prevention (Hainan Academy of Preventive Medicine), Haikou, China

**Keywords:** Molecular biology, Immunology, Cell biology

## Abstract

*Plasmodium*-exported proteins bind with membrane receptors of host red blood cell to play the canonical role in erythrocyte invasion. While their potential to interact with immune cell receptors and orchestrate inflammatory responses remains largely unexplored. Among these proteins, tryptophan-rich antigens (TRAgs) participate in invading erythrocytes and demonstrate strong immunogenicity in murine malaria models. Here, we demonstrate that *Plasmodium yoelii* (Py) TRAg7 interacts with the macrophage receptor cluster of differentiation 71 (CD71) and activates nuclear factor kappa B p65 signaling pathway, which promoted the production of proinflammatory factors. Consistently, genetic deletion of TRAg7 led to decreased parasitemia, reduced inflammation, and improved host survival. Collectively, these findings reveal that exported proteins drive the inflammatory response by binding to macrophage receptor, expanding the current knowledge of the biological effects of malaria parasite-host interaction beyond erythrocyte invasion. This study offers a conceptual framework for better understanding malaria pathogenesis and potential therapeutic intervention strategies.

## Highlights

• *Plasmodium* tryptophan-rich antigen 7 (TRAg7) bound to macrophages in the spleen
• TRAg7 interacted with the macrophage membrane receptor CD71
• TRAg7 enhanced the production of proinflammatory cytokines via NF-κB signaling
• TRAg7 deficiency reduced the parasitemia and improved the survival in rodent model

## Graphical abstract

**Figure:**
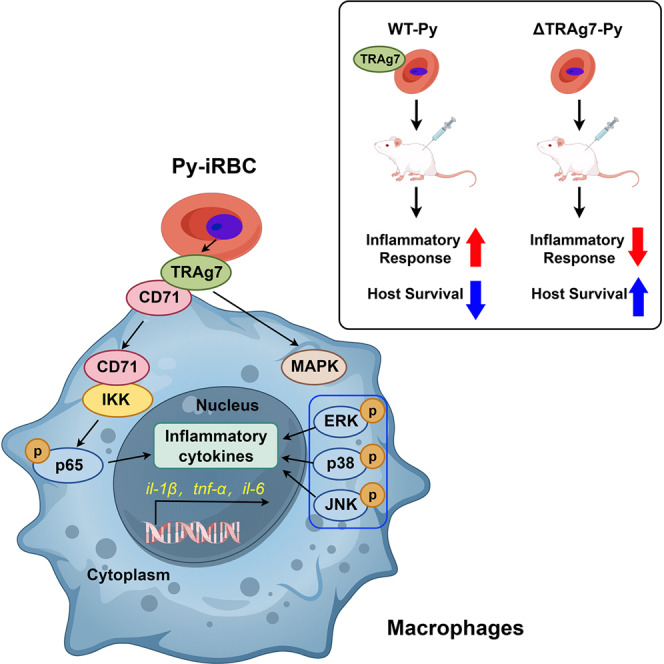

## Introduction

Malaria caused an estimated 597,000 deaths in 2023.^1^ Complex interactions between malaria parasites and the host disrupt immune regulation, contributing to disease pathogenesis and parasite proliferation. The spleen is essential for generating effective immune responses and limiting parasitemia during infection.^2^ As parasites multiply, infected red blood cells (RBCs) rupture and release heme, parasite DNA, and other antigens. These pathogen-associated molecular patterns activate host immune defenses.^3^^,^^4^ However, this response is often dysregulated and insufficient to clear the infection. Hyperactivation of the immune system and excessive production of proinflammatory cytokines contribute to immunopathology and tissue damage.^5^^,^^6^^,^^7^

The spleen plays a central role in malaria immunity by sensing and clearing parasitized RBCs and orchestrating innate and adaptive immune responses, as well as erythropoiesis.^8^ Malaria infection induces a strong inflammatory response in the spleen, including macrophage recruitment.^8^ In a murine model, *Plasmodium yoelii* (Py) sporozoites and their lysates strongly activated Toll-like receptor 2 on macrophages and induced proinflammatory cytokines such as interleukin-6 (IL-6), monocyte chemoattractant protein-1, and tumor necrosis factor-α (TNF-α), thereby inhibiting erythrocytic parasite growth.^9^ Macrophages therefore represent a key component of the innate immune response to malaria.^10^ Although macrophages contribute to host defense, macrophage activation is also associated with adverse clinical outcomes.^11^ Severe malaria is characterized by excessive inflammatory cytokine production, systemic inflammation, and vascular dysfunction.^12^^,^^13^ Macrophages and monocytes are major sources of elevated proinflammatory cytokines in patients with severe malaria.^14^ Interactions between macrophages and malaria parasites thus influence the balance between protective immunity and immunopathology.^11^ For example, interaction of macrophages with the *Plasmodium falciparum* erythrocyte membrane protein 1 (PfEMP1) suppresses IL-1β and IL-6 production, illustrating a potential mechanism for immune suppression by *P*. *falciparum*.^15^

Hundreds of *Plasmodium* proteins are exported to the RBC surface and are referred to as *Plasmodium* exported proteins. These proteins are critical for RBC invasion and immune modulation.^16^^,^^17^ The *Plasmodium berghei* homolog of the human T cell immunomodulatory protein is expressed on the merozoite surface and exported to the erythrocyte membrane during infection; it then binds to macrophages and suppresses host inflammatory responses.^17^ These findings suggest that *Plasmodium* exported proteins play important roles in macrophage interaction and immune modulation. Tryptophan-rich antigens (TRAgs) in malaria parasites participate in host cell invasion and signal transduction events.^18^^,^^19^^,^^20^ TRAgs were first reported in Py, where PypAg-1 (referred to as PyTRAg2 in this study) contains a tryptophan-rich region and localizes both in the cytoplasm and on the surface of Py-infected RBCs (iRBCs).^21^ Homologous members of the TRAg family identified in *Plasmodium vivax* (Pv) bind to host erythrocytes through multiple receptors.^19^ Memory T cells specific for PvTRAgs containing conserved sequences have been detected in individuals exposed to Pv malaria.^20^ PvTRAg antigens elicit strong humoral immune responses, generating high titers of acquired antibodies in patients with Pv infection.^20^^,^^22^^,^^23^ Naturally occurring anti-PvTRAg antibodies can aid in the diagnosis of Pv infection and help distinguish it from infections caused by other *Plasmodium* species.^24^ PvTRAgs have therefore been considered promising candidates for vaccine development and diagnostic applications. Additionally, PvTRAgs bind to human splenic fibroblasts, activate inflammatory signaling pathways, and regulate collagen secretion.^25^ Previous investigations of TRAgs have largely focused on antigenicity, immunogenicity, and the identification of RBC-binding proteins.^26^ However, the role of TRAgs in modulating macrophage-mediated immune responses and their underlying molecular mechanisms needs to be thoroughly elucidated.

In this study, we found that among members of the PyTRAg family, only TRAg7 bound to spleen cells and macrophages. TRAg7 activated the mitogen-activated protein kinase (MAPK) and nuclear factor-kappa B (NF-κB) p65 pathways and increased the production of proinflammatory cytokines in macrophages. Furthermore, TRAg7 interacted with cluster of differentiation 71 (CD71) on the macrophage surface, thereby inducing inflammatory responses. In a murine malaria model infected with Py17XL, genetic deletion of *trag7* significantly reduced parasitemia, improved host survival, and lowered circulating proinflammatory cytokine levels. As a TRAg family member with conserved sequences, TRAg7 promotes inflammatory activation in macrophages by binding to CD71 and contributes to malaria pathogenesis. This study demonstrates the critical role of TRAg proteins in regulating macrophage inflammatory responses and elucidates the underlying molecular mechanism. Our data transcend the identification the interplay between parasites and host macrophages. They reveal that *Plasmodium* parasites engage in an interaction with host through the exported proteins to macrophages membrane receptors beyond canonical pattern recognition receptors, to activate inflammatory pathways. These findings provide a revised framework for understanding how parasites induce excessive inflammation, leading to severe immunopathology, with potential implications for the development of targeted therapeutics.

## Results

### *Plasmodium yoelii* TRAg7 binds to macrophages

**Figure 1:**
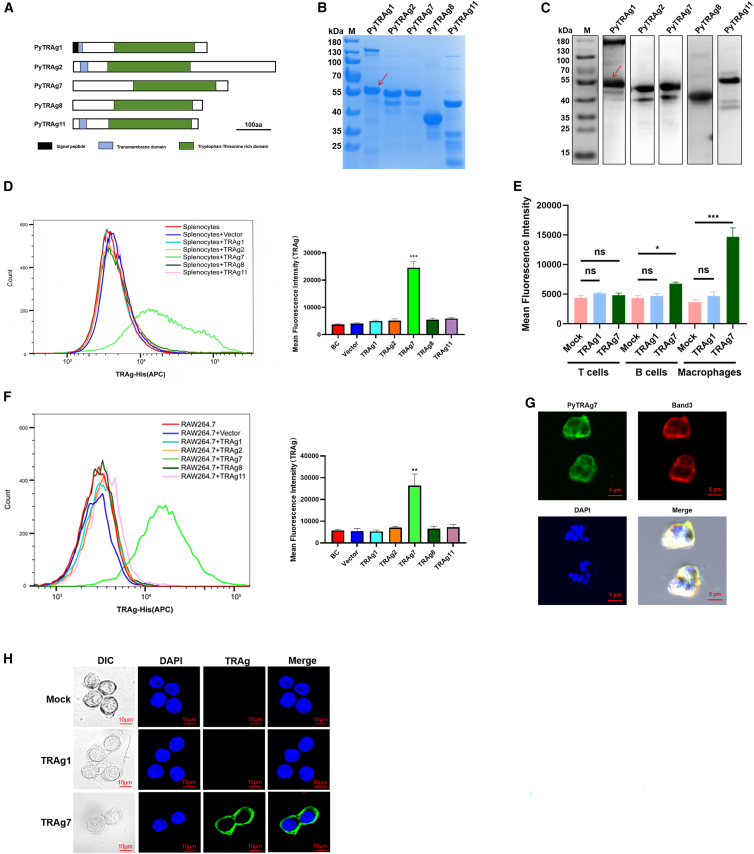
PyTRAg7 bound to the macrophages (A) Schematic diagram of the primary structure of PyTRAgs proteins. Blue boxes indicate the transmembrane domains. Black boxes indicate the signal peptides. The tryptophan-threonine-rich domain is presented in the green boxes. Scale bars, 100 amino acids (aa). (B and C) The recombinant proteins containing His-tag of PyTRAgs were separated by SDS-PAGE gels, stained with Coomassie brillant blue (B), and verified by the western blotting with anti-His-Tag antibody (C). M, marker. (D) PyTRAg1, PyTRAg2, PyTRAg7, PyTRAg8, PyTRAg11, and vector (40 μg) recombinant proteins were incubated with 2 × 10^6^ spleen cells at room temperature (RT) for 2 h, His-APC-labeled spleen cells were detected by flow cytometry (left). Quantitative analysis of MFI (right). BC (blank control) indicated splenocytes only. (E) Quantitative analysis of the binding ability of recombinant protein PyTRAg7 to primary spleen macrophages, T cells and B cells were determined using flow cytometry by MFI. PyTRAg1 protein as the negative control. (F) A total of 40 μg of PyTRAg1, PyTRAg2, PyTRAg7, PyTRAg8, PyTRAg11, and vector proteins were incubated with RAW264.7 cells at RT for 2 h. Cells were washed and stained with APC-His antibody, and the binding ability of PyTRAgs to cells was determined using flow cytometry (left). Quantitative analysis of MFI (right). BC (blank control) indicated RAW264.7 cells only. (G) The localization of PyTRAg7 in iRBC was analyzed using the immunofluorescence assay. The primary antibodies were anti-PyTRAg7 mouse antibodies and anti-Band3-Loop5 (extracellular region of Band3) rabbit antisera. The secondary antibodies used were goat anti-mouse 488 fluorescent and donkey anti-rabbit 568 fluorescent antibodies. Scale bars, 5 μm. (H) Immunofluorescence analysis of the localization of PyTRAg7 in RAW264.7 cells. Scale bars, 10 μm. Data are represented as mean ± SEM. One-way ANOVA was used to compare multiple groups of samples, and the SNK test was used for pound-for-pair comparison. (ns, no significance; ∗*p*＜0.05，∗∗*p*＜0.01，∗∗∗*p*＜0.001).

Five homologous proteins of the PvTRAg family, PyTRAg1, PyTRAg2, PyTRAg7, PyTRAg8, and PyTRAg11, were identified in Py17XL using the PlasmoDB database (http://plasmodb.org/plasmo). These proteins share a characteristic tryptophan-rich domain in the C-terminal region (Figure 1A). PyTRAgs with His-tag were successfully expressed using an *Escherichia coli* (*E*. *coli*) expression system, purified by nickel column-affinity chromatography, and identified by Coomassie brilliant blue (Figure 1B). All polyclonal antibodies generated against the recombinant PyTRAgs recognized the corresponding natural PyTRAg proteins in the lysates of iRBCs (Figure 1C). The purities of the recombinant PyTRAg1, PyTRAg2, PyTRAg7, PyTRAg8, and PyTRAg11 proteins were 90, 80, 85, 95, and 80%, respectively. The final protein concentrations were 2, 1.4, 1, 3, and 1.6 mg/mL, respectively.

Given that TRAgs are parasite-exported proteins and the spleen is a key organ mediating host-*Plasmodium* interactions and regulating immune responses, we investigated whether PyTRAgs can bind to splenocytes. Recombinant PyTRAg proteins were co-incubated with mouse splenocytes. Among the PyTRAg protein family, only TRAg7 showed a significant increase in both the number of bound cells and binding intensity (Figure 1D). To identify the specific splenic cell populations that interact with TRAg7, splenocytes were incubated with PyTRAg7, followed by detection of cell types by flow cytometry. Although macrophages represent a relatively small proportion of spleen cells, their mean fluorescence intensity (MFI) after TRAg7 incubation was significantly higher than that of T cells or B cells (Figure 1E; Figure S1). The binding of TRAg7 protein to macrophages was significantly stronger than that in the untreated control. In contrast, the negative control protein TRAg1 showed minimal binding to T cells, B cells, and macrophages (Figure 1E; Figure S1). These findings indicate that PyTRAg7 is the only member of the TRAg family that binds to primary macrophages in spleen.

Macrophages constitute the first line of defense against *Plasmodium* parasites and play a central role in regulating splenic immune responses. To determine whether PyTRAgs directly bind to macrophages, recombinant PyTRAgs proteins were co-incubated with RAW264.7 murine macrophage cell line. PyTRAg7-treated cells showed significantly higher MFI than the other four PyTRAgs, indicating that only PyTRAg7 binds directly to macrophages (Figure 1F). To evaluate the localization of PyTRAg7, indirect immunofluorescence assays (IFAs) using a mouse antibody against PyTRAg7 and a rabbit antibody against Band3-Loop5 (extracellular region of Band3) demonstrated that PyTRAg7 is present on the surface of iRBCs (Figure 1G). After co-incubation, PyTRAg7 colocalized with the macrophage membrane, suggesting that it interacts with surface molecules on macrophages (Figure 1H). In addition, PyTRAg7 also promoted the proliferation of splenocyte and primary macrophages n a concentration-dependent manner (Figures S2A and S2B). These results imply that only PyTRAg7 binds to macrophages among the TRAgs protein family.

### PyTRAg7 promotes the production of inflammatory factors of macrophages

**Figure 2:**
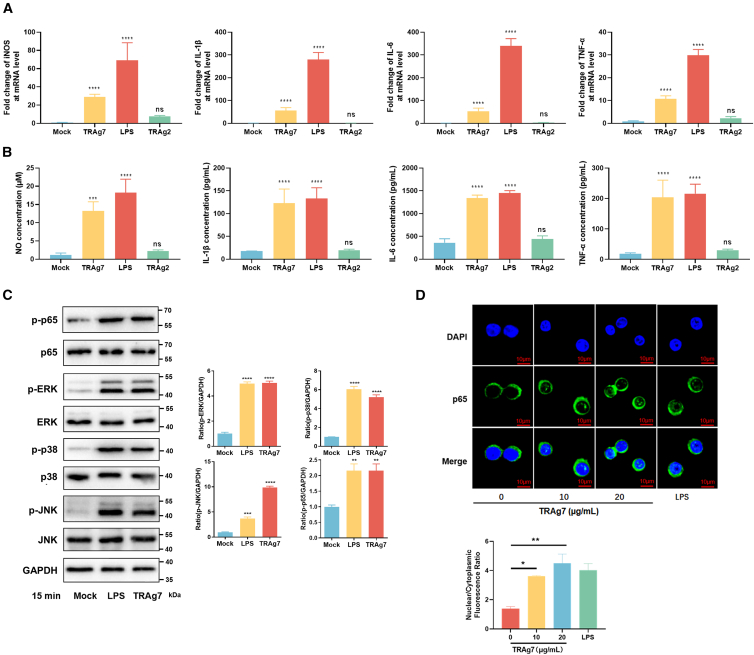
TRAg7 promoted macrophages to produce proinflammatory cytokines via activation of the NF-κB and MAPK signaling pathways (A) The mRNA level of iNOS, IL-1β, IL-6, and TNF-α in RAW264.7 cells after PyTRAg7 or PyTRAg2 recombinant protein (10 μg/mL) treatment for 48 h, as detected by RT-qPCR. GAPDH was used as an internal control. LPS was used as the positive control. (B) The protein level of NO, IL-1β, IL-6, and TNF-α produced by RAW264.7 cells after PyTRAg7 or PyTRAg2 recombinant protein (10 μg/mL) treatment for 48 h, as detected by ELISA. LPS was used as a positive control. (C) RAW264.7 cells were treated with 10 μg/mL PyTRAg7 for 15 min. The protein expression of p-p65, p65, p-ERK, ERK, p-p38, p38, p-JNK, and JNK in RAW264.7 cells was detected by western blotting. LPS was used as a positive control. Protein levels of p-ERK, p-p38, p-JNK and p-p65 were semi-quantified and normalized to GAPDH (right). (D) Nuclear translocation of the p65 subunit in RAW264.7 cells was determined by immunofluorescence. LPS was used as a positive control. Quantitative analysis of nuclear translocation of p65. Scale bars, 10 μm. Data are represented as mean ± SEM. One-way ANOVA was used to compare multiple groups of samples, and the SNK test was used for pound-for-pair comparison. (ns, no significance; ∗*p <* 0.05, ∗∗*p* < 0.01, ∗∗∗*p* < 0.001, ∗∗∗∗*p* < 0.0001).

We next examined the effects of TRAg7 on proinflammatory cytokine production by macrophages. Compared with untreated cells, TRAg7 induced RAW264.7 to produce significantly higher mRNA and protein levels of proinflammatory cytokines, including IL-6, IL-1β, and TNF-α. TRAg7 promoted the inducible nitric oxide synthase (iNOS) expression and nitric oxide (NO) production in murine macrophage cell line RAW264.7 (Figures 2A and 2B). It is worth noting that TRAg1 could also elevate the levels of proinflammatory cytokines TNF-α and IL-6 in macrophages (Figure S3). Thus we used TRAg2 as the negative control to the effect of TRAg7 on the expression of inflammatory factors in macrophages. No significant differences in cytokine production were observed between untreated RAW264.7 and cells treated with TRAg2 (Figures 2A and 2B). Taken together, these results indicate that PyTRAg7 enhances the inflammatory response of macrophages.

During malaria infection, the production of inflammatory cytokines is primarily dependent on activation of the MAPK and NF-κB p65 signaling pathways.^6^ We therefore examined whether PyTRAg7 induces inflammatory factor expression through these pathways. PyTRAg7-treated macrophages showed significant increases in the phosphorylation of p38, ERK, and JNK (Figure 2C). PyTRAg7 also upregulated phosphorylation of NF-κB p65 (Figure 2C) and promoted nuclear translocation of the p65 subunit in a dose-dependent manner (Figure 2D). Taken together, PyTRAg7 binds to macrophages, enhances macrophage proliferation, promotes M1 polarization, and activates downstream MAPK and NF-κB p65 signaling pathways, leading to the production of NO and the proinflammatory cytokines IL-1β, IL-6, and TNF-α.

### PyTRAg7 binds to the macrophage membrane receptor CD71

**Figure 3:**
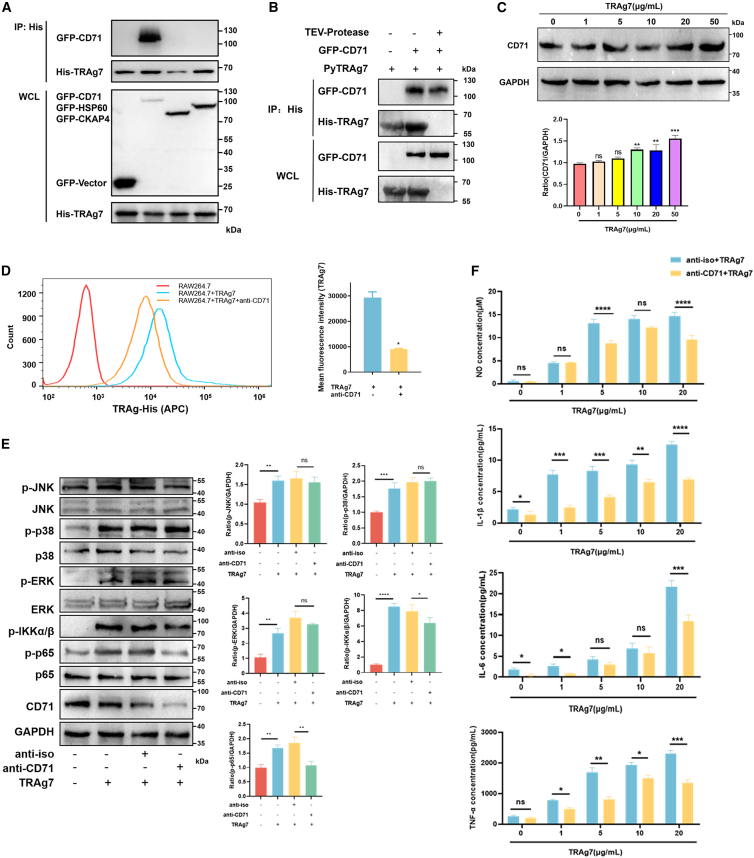
PyTRAg7 bound to macrophage membrane protein CD71 (A) The pEGFP-C1-vector, pEGFP-CD71, pEGFP-CKAP4, and pEGFP-HSP60 plasmids were transfected into HEK293T cells for 24 h. The cell lysate was incubated with PyTRAg7 recombinant protein containing His-Tag (200 μg/mL) on Ni-NTA resin at 4 °C overnight. After washing the Ni-NTA resin, the eluted protein was subjected to western blotting and eluted to verify the binding protein. (B) Recombinant PyTRAg7 was cleaved with His‑tagged TEV protease (4°C, 12 h), and the His‑tagged protease together with uncleaved proteins were removed using Ni‑NTA resin to obtain tag‑free PyTRAg7. HEK293T cell lysates expressing pEGFP‑CD71 were incubated with either His‑tagged or tag‑free PyTRAg7 (200 µg/mL) and subjected to pull‑down analysis. (C) RAW264.7 cells were stimulated with PyTRAg7 recombinant protein at the indicated concentrations for 48 h. Indicated proteins were detected by the western blotting. Protein levels of CD71 were semi-quantified and normalized to GAPDH. (D) RAW264.7 cells were pretreated with or without rabbit anti-CD71 antibody for 2 h and stimulated with 10 μg/mL PyTRAg7 recombinant protein for 20 min at RT. Cells were incubated with APC-His antibody for 2 h on ice, washed, and identified using flow cytometry. (E) RAW264.7 cells were pretreated with or without rabbit anti-CD71 antibody for 2 h and stimulated with PyTRAg7 recombinant protein (10 μg/mL) for 15 min. Indicated proteins were detected by the western blotting. Protein levels of p-ERK, p-p38, p-JNK, p-IKKα/β, and p-p65 were semi-quantified and normalized to GAPDH (right). (F) The NO production and the protein level of IL-1β, IL-6, and TNF-α in RAW264.7 cells supernatant after PyTRAg7 treatment with or without anti-CD71 antibody for 2 h, as detected by ELISA. Data are represented as mean ± SEM. One-way ANOVA was used to compare multiple groups of samples, and the SNK test was used for pound-for-pair comparison. (ns, no significance; ∗*p* < 0.05, ∗∗*p* < 0.01, ∗∗∗*p* < 0.001, ∗∗∗∗*p* < 0.0001).

We further identified the macrophage receptor responsible for PyTRAg7 binding. His-tagged PyTRAg7 was immobilized on affinity resin and incubated with macrophage membrane protein extracts. Candidate interacting membrane proteins were identified by silver staining and liquid chromatography-mass spectrometry (LC-MS) (Table 1). Pull-down assays demonstrated that PyTRAg7 specifically bound to CD71, but not to heat shock protein 60 (HSP60) or cytoskeleton-associated protein 4 (CKAP4), which were also detected in membrane fractions (Figure 3A). Although His-tag was removed from the PyTRAg7 protein using TEV protease, PyTRAg7 still bound directly to CD71 by pull-down assay (Figure 3B). PyTRAg7 promoted the expression of CD71 in RAW 264.7 cells in a dose-dependent manner (Figure 3C). To confirm that CD71 serves as the macrophage receptor for PyTRAg7, we blocked CD71 on the macrophage surface using anti-CD71 antibody, and verified the neutralization effect by inhibition assay (Figure S4A). After coincubation with PyTRAg7, CD71 blockade significantly reduced the number of fluorescently labeled macrophages, indicating that inhibiting CD71 diminished PyTRAg7 binding (Figure 3D). These findings support CD71 as the macrophage receptor for PyTRAg7. Following CD71 blockade, there were no significant changes in the phosphorylation of JNK, p38, or ERK (Figure 3E). In contrast, phosphorylation of inhibitor κB kinase α/β (IKKα/β) and NF-κB p65 was significantly decreased (Figure 3E). Consistent with these signaling changes, anti-CD71 treatment reduced the production of NO and the proinflammatory cytokines IL-1β, IL-6, and TNF-α (Figure 3F). As CD71 is also expressed on the surface of RBCs, we further showed that PyTRAg7 can bind to CD71 on the surface of RBCs (Figure S4B). Together, these results indicate that PyTRAg7 activates the NF-κB p65-signaling pathway and promotes NO and proinflammatory cytokines by binding to the macrophage receptor CD71.

**Table 1:** Identification of macrophage membrane proteins binding to PyTRAg7

No.	Protein name	Accession number	Coverage (%)	Mol. wt (kDa)	Unique peptides
1	Cytoskeleton-associated protein 4 (CKAP4)	Q8BMK4	14.78	63.7	16
2	60 kDa heat shock protein (HSP60)	P63038	22.51	60	11
3	Transferrin receptor protein 1 (CD71)	Q62351	4.08	85.7	10
4	Formin-like protein 1	Q9JL26	4.94	122.1	5

### PyTRAg7 is required for parasite growth during the blood stage

**Figure 4:**
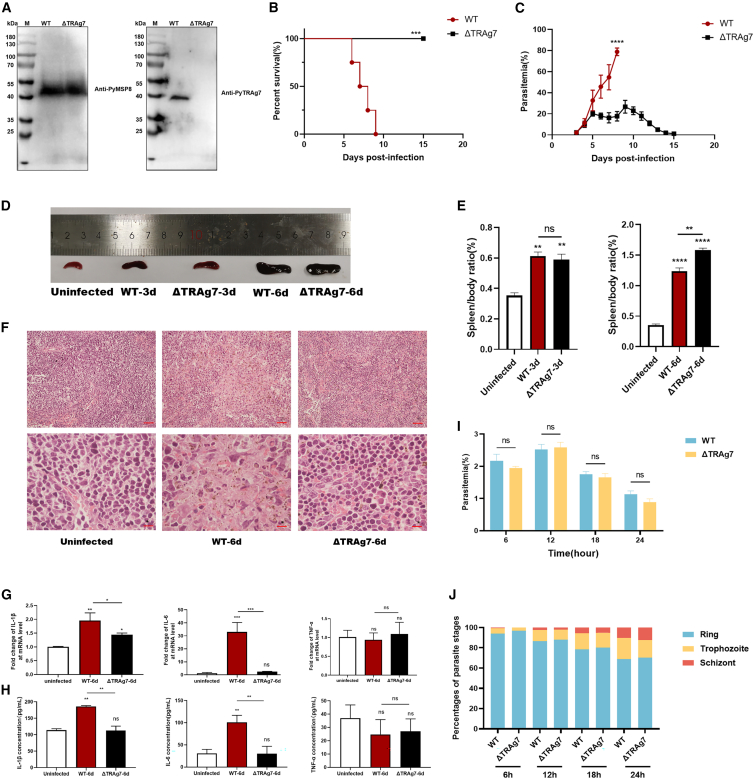
PyTRAg7-deletion strain infection improved the host survival and downregulated the proinflammatory cytokines (A) PyWT and ΔTRAg7 strain were lysed and identified by western blotting with anti-TRAg7 and anti-MSP8 antisera. (B) Survival rates of mice after WT or ΔTRAg7 strain infection for 0–15 days. (C) Daily parasitemia of mice after WT or ΔTRAg7 strain infection for 0–15 days. (D) The gross pathology of spleens of mice infected with WT and ΔTRAg7 at three- and six-days post-infection. (E) The spleen/body weight of mice infected with WT or ΔTRAg7 parasites at three- and six-days post-infection. (F) HE staining of spleen in mice infected with WT and ΔTRAg7 at six days post-infection. The top scale bars are 50 μm. The bottom scale bars are 10 μm. (G) The mRNA levels of IL-1β, IL-6, and TNF-α in the spleen of mice infected with WT and ΔTRAg7 strain were detected by RT-qPCR at six days post-infection. GAPDH was used as an internal control. (H) Serum levels of IL-1β, IL-6, and TNF-α were measured using ELISA at six days post-infection in WT and ΔTRAg7 infected mice. (I) Parasitemia over time after *in vitro* invasion of the schizonts extracted from WT and ΔTRAg7 infected mice. (J) Percentages of ring, trophozoite, and schizont stages at different time points after invasion. Data are from *n* = 6–8 mice per group for (B–H). Data are represented as mean ± SEM. One-way ANOVA was used to compare multiple groups of samples, and the SNK test was used for pound-for-pair comparison. Student’s *t* test was used to compare the two groups of independent samples. (ns, no significance; ∗*p* < 0.05, ∗∗*p* < 0.01, ∗∗∗*p* < 0.001, ∗∗∗∗*p* < 0.0001).

To evaluate the role of TRAg7 in parasite growth and inflammatory responses during the blood stage *in vivo*, we generated a *Trag7* gene-deletion Py mutant (ΔTRAg7) (Figure S5A). PCR further confirmed the successful deletion of *Trag7* (Figure S5B). Deletion of PyTRAg7 was confirmed at the protein level. Native PyTRAg7 protein was undetectable in the ΔTRAg7 strain-infected RBCs, whereas the control natural Py merozoite surface protein 8 (MSP8) was detected in both WT- and ΔTRAg7-infected RBCs, confirming that *Trag7* deletion strain was successfully constructed (Figure 4A). WT-infected BALB/c mice died from hyperparasitemia 6–9 days post-infection, while all ΔTRAg7-strain infected mice survived (Figure 4B). ΔTRAg7-infected mice exhibited significantly lower parasitemia, which declined after reaching a peak of 30% and ultimately cleared the parasites completely (Figure 4C).

The spleen is a key immune organ in acute *Plasmodium* infection, and splenomegaly is a common manifestation of malaria. These data demonstrate that TRAg7 binds to splenocytes. Accordingly, we evaluated the effect of TRAg7 on the gross and histopathological features of the spleen in a murine malaria model. The gross morphology of spleens from WT- and ΔTRAg7-infected mice appeared similar (Figure 4D). However, at 6 days post-infection, the spleen/body weight ratio was significantly higher in ΔTRAg7-infected mice than in WT-infected mice (Figure 4E), and spleens from ΔTRAg7-infected mice remained structurally intact (Figure 4F). These findings indicate that splenomegaly was more pronounced in ΔTRAg7-infected mice than in WT-infected mice. PyTRAg7 upregulates proinflammatory factors in macrophages. We therefore assessed the inflammatory response *in vivo*. At 6 days post-infection, IL-6 and IL-1β levels in the spleen and serum of ΔTRAg7-infected mice were significantly lower than in those of WT-infected mice (Figures 4G and 4H). Loss of TRAg7 attenuated acute inflammatory responses, reduced parasitemia, and prolonged survival in the murine malaria model.

To investigate whether the *Trag7* gene could affects the invasion of RBCs by parasites without impacting the host immune responses, we conducted an *in vitro* invasion experiment. There was no significant difference in the parasitic levels of WT and ΔTRAg7 cultures at different time points (Figure 4I). The percentage of schizonts increased over time, but there was no significant difference between WT and ΔTRAg7 strain (Figure 4J). These results indicate that the TRAg7 gene does not significantly alter the invasion of RBCs *in vitro*.

## Discussion

Malaria infection elicits severe immune activation and inflammatory cytokine storms, which influence both disease progression and clinical outcomes.^27^ The spleen serves as the central organ regulating immune responses during malaria.^2^^,^^8^ *Plasmodium* export proteins are essential in shaping proinflammatory cytokine production during pathogen-host interactions.^15^^,^^28^^,^^29^ In this study, we characterized the role of the *Plasmodium* export protein TRAg7 in initiating innate immune responses. TRAg7 bound to macrophages through the CD71 receptor, activated the NF-κB p65 signaling pathway, and triggered sustained release of proinflammatory mediators. Deletion of TRAg7 in the lethal Py17XL strain significantly increased host survival, likely through reduced proinflammatory cytokine production *in vivo*. These results demonstrate that PyTRAg7 exacerbates host inflammatory response by binding to the cell receptor CD71 in macrophages, contributing to pathogenesis in the rodent malaria model. These findings advance the understanding of how *Plasmodium* export proteins regulate inflammatory responses and highlight mechanisms governing pathogen-host interactions in malaria.

Inflammation is a central component of malaria-associated pathology. During blood-stage infection, pathogen-associated molecular patterns derived from the parasite engage pattern-recognition receptors on host cells, initiating innate immune responses and producing proinflammatory cytokines.^27^^,^^30^ *Plasmodium* parasites activate host monocytes and macrophages, which subsequently produce inflammatory cytokines associated with severe malaria.^14^^,^^30^ Importantly, *in vitro* binding assays demonstrated that among the TRAg family proteins analyzed in this study, only TRAg7 exhibited a direct and significant binding capacity to murine macrophages. Because mature erythrocytes lack intrinsic trafficking machinery, the parasite has evolved a specialized secretory system to transport proteins into and through the host cell cytoplasm.^31^ Exported proteins are mainly classified based on the presence or absence of the *Plasmodium* export element or PEXEL motif.^16^^,^^32^ Since the amino acid sequence of PyTRAg7 does not contain the characteristic RxLxE/D/Q motif, PyTRAg7 is likely a PEXEL-negative exported protein. As an exported protein, PyTRAg7 may contribute to host cell remodeling. This suggests that TRAg7 may play a unique role in mediating parasite-macrophage interactions compared to the other TRAg family members. It is important to note that the other Py antigen PySRA (surface-related antigen) has been shown to modulate host proinflammatory responses by binding to CD68 on the macrophage membrane.^29^ We further demonstrated that PyTRAg7 increased expression of iNOS and proinflammatory cytokines IL-1β, IL-6, and TNF-α (Figures 2A and 2B). Because the MAPK and NF-κB pathways play pivotal roles in macrophage inflammatory signaling, we investigated these pathways. PyTRAg7 significantly increased phosphorylation of p38, ERK, and NF-κB p65, and facilitated nuclear translocation of NF-κB p65 (Figure 2D), thereby activating the MAPK- and NF-κB-signaling pathways. *Plasmodium* SRA and PfEMP1 modulate macrophage inflammatory responses through the NF-κB pathway.^15^^,^^29^ This was consistent with our findings and supports the conclusion that *Plasmodium* export proteins play important immunomodulatory roles in pathogen-host macrophage interactions.

Next, we hypothesized that specific membrane molecules on macrophages are required for PyTRAg7-induced proinflammatory responses. To investigate this, TRAg7-associated macrophage membrane proteins were separated by SDS-PAGE and identified by MS. Several potential interacting proteins were detected (Table 1). Pull-down assays showed a strong interaction between PyTRAg7 and CD71 (Figures 3A and 3B), indicating that CD71 has a high affinity for PyTRAg7 and functions as its macrophage receptor. CD71, also known as transferrin receptor 1 (TfR1), contains two key functional regions. The N-terminal region (1–67 aa) mediates interaction with SH3BP4 and regulates internalization of the transferrin receptor.^33^ The C-terminal ectodomain (569–760 aa) serves as the ligand-binding domain. Based on domain localization and functional characteristics, we speculate that PyTRAg7 likely interacts with the ligand-binding ectodomain of CD71. Consistent with this, anti-CD71 treatment markedly reduced PyTRAg7 binding to macrophages and significantly inhibited activation of the NF-κB p65 pathway and the production of the proinflammatory cytokines IL-1β, IL-6, and TNF-α (Figures 3E and 3F). Accordingly, we observed reduced levels of phosphorylated IKK after CD71 blockade (Figure 3E). The data from the single-cell transcriptome database indicates that CD71 is mainly expressed in monocytes and macrophages in the spleen.^34^ Monocytes are the precursor cells of macrophages, and macrophages are tissue resident cells in spleen and play a crucial role in inflammatory responses. This further explains the finding of this study that the TRAg7 protein can bind to CD71 in macrophages. In idiopathic pulmonary fibrosis, CD71-expressing alveolar macrophages display enhanced phagocytic capacity and upregulate activation-associated surface proteins such as CD86, suggesting that CD71 contributes to macrophage activation.^35^ Interestingly, as a *Plasmodium* export protein, PyTRAg7 binds to CD71 on the surface of RBCs (Figure S4B), suggesting that it may also play a crucial role in RBC invasion during *Plasmodium* infection, especially in the invasion of reticulocytes, although further validation is required.

Mechanically, CD71 is an IKK-interacting protein in U2OS osteosarcoma and HEK293 cells, and its depletion inhibits IKK complex formation and NF-κB-dependent transcription.^36^ Our findings show that PyTRAg7 binds CD71 on the surface of macrophages and regulates NF-κB-mediated inflammatory response (Figure 3). Therefore, we speculate that TRAg7 influences NF-κB-dependent cytokine production by modulating the interaction between CD71 and the IKK complex. Our results showed that neutralization of CD71 did not significantly impact the phosphorylation of JNK or ERK, which suggested that the regulation of macrophage inflammatory response is not entirely dependent on the CD71 receptor. Previous studies have revealed that *P*. *falciparum* erythrocyte membrane protein 1 (PfEMP1) could bind to multiple receptors of the host cells, including CD36 and ICAM-1, and plays a crucial role in the pathogenesis of malaria.^37^

Notably, sustained activation of JNK/ERK pathway upon CD71 blockade indicated that CD71-independent signaling branches. We propose that in addition to CD71, TRAg7 may engage multiple receptors leading to downstream effects. Future work could further identify additional TRAg7-binding partners on macrophages and detected the signal activation by simultaneously blocking. Interestingly, previous study showed that JNK/ERK pathway are also functionally redundant for the expression of TNF-α and IL-6 induced by *Plasmodium* glycosylphosphatidylinositols in macrophages.^38^ Therefore, we believe that this signaling redundancy may be biologically important in ensuring robust immune activation during malaria. As Pv preferentially invades CD71^+^ immature reticulocytes,^39^ we further speculate that CD71 serves as a reticulocyte receptor for PvTRAg family proteins involved in erythrocyte invasion. Antibody-blocking assays only partially inhibited CD71 and did not eliminate its expression or function. To conclusively determine whether CD71 is the primary receptor for PyTRAg7, its role in PyTRAg7 binding should be more definitively assessed by examining PyTRAg7 binding in CD71-knockout cells in future studies.

To further evaluate the role of PyTRAg7 in the rodent malaria model, clustered regularly interspaced short palindromic repeats (CRISPR)-CRISPR associated 9 (Cas9) gene editing was used to delete the full-length PyTRAg7 (Figure 4A; Figure S5A). Infection with the PyTRAg7 deletion strain improved host survival (Figure 4B). The ΔTRAg7 strain proliferated at a substantially lower rate than the WT strain in peripheral blood (Figure 4C). A previous study reported that the tryptophan-rich domain-containing proteins IPIS2 and IPIS3 are required for efficient blood-stage growth,^40^ which is consistent with our findings. Two mechanisms may explain the improved survival of hosts infected with the TRAg7 deletion strain. First, when compared with WT-infected mice, ΔTRAg7-infected mice exhibited enlarged but structurally intact spleens (Figures 4D and 4E), which may enhance clearance of infected erythrocytes. Second, the levels of inflammatory cytokines were markedly reduced in ΔTRAg7-infected hosts at six days post-infection (Figures 4G and 4H), thereby protecting the host from excessive inflammation. A recent review emphasized that host protection is achieved only when inflammatory responses and antiparasitic immunity remain balanced.^7^ An excessively strong inflammatory response in the host can trigger a cytokine storm, leading to immune imbalance and pathological damage, thus reducing survival. Patients with severe malaria commonly exhibit high levels of IL-1β, IL-6, and TNF-α.^14^ Given that IL-6 has been proven to exert immunosuppressive effects in the later stages of *Plasmodium* infection^41^ and that the IL-6 level in the TRAg7-deficient parasite strain infected mice was significantly lower than that in the WT group (Figures 4G and 4H), using the injection of recombinant IL-6 restored its function can further explore the mechanisms of TRAg7-induced immunopathological damage. Moreover, it is necessary to use ΔTRAg7 strain to infected the myeloid-specific CD71 knockout mice, to further determine the whether CD71 of macrophages contribute to the effects of TRAg7 knockout parasite strains on parasitemia and host survival rate.

We observed the improved survival and reduced parasitemia with the ΔTRAg7 parasite, despite unchanged intrinsic growth *in vitro*, suggesting that TRAg7 altered host-parasite interactions rather than a proliferation defect. We speculate that this might be due to the following underlying mechanism. Firstly, immune-mediated effects may include that TRAg7 promoted the cytokines production such as IL-6, to induce proinflammatory monocytes to inhibit immunity.^41^ Secondly, enhanced mechanical clearance by the spleen due to high parasitemia, thus increasing the parasites clearance. In addition, the deletion of TRAg7 may regulate the functions of other parasite proteins, thereby affecting the erythrocyte tropism. This multifactorial framework underscores that the *in vivo* phenotype is a nuanced integration of vascular biology, innate filtration, and adaptive immunity, rather than a purely immune-driven outcome.

Tryptophan-rich domains contain conserved tryptophan residues, and proteins with this domain play essential roles in host-parasite interactions. Several PvTRAg family members bind erythrocyte receptors and influence parasite growth and invasion.^19^^,^^26^ PvTRAg38 interacts with the erythrocyte receptors Band3 and Basigin, promoting parasite development.^26^^,^^42^^,^^43^ Some PvTRAg proteins share erythrocyte receptors, providing a theoretical foundation for therapeutic approaches targeting Pv infection.^19^^,^^42^ PvTRAg23 binds to human splenic fibroblasts through the receptor vimentin and modulates inflammatory responses.^28^ Loss of the tryptophan-rich protein IPIS2 impairs the ability of infected erythrocytes to bind endothelial cell CD36, indicating that TRAg family members are important for efficient sequestration of schizonts.^40^ Our findings showed that PyTRAg7 interacts with macrophages through the surface molecule CD71, promoting inflammatory responses (Figure 3). This aligns with previous work demonstrating that PySRA enhances the production of proinflammatory cytokines by binding to macrophage surface protein CD68.^29^ In addition to erythrocyte membranes, TRAg proteins may also be present within parasite-derived extracellular vesicles, participating in host-pathogen interactions.^44^ Previous studies mainly focused on *Plasmodium* exported proteins binding to the RBC membrane proteins, which play a crucial role in the erythrocyte invasion.^42^^,^^43^ However, an increasing amount of evidence indicates that the exported proteins could also interact with other surface molecules on the host cells to regulate inflammatory responses and cell adhesion, thereby participating in the malaria pathogenesis. Collectively, the evidence indicates that TRAg family members participate in interactions between multiple host cells and parasites, suggesting that they have diverse roles in malaria pathogenesis. These properties also highlight the potential of TRAgs as targets for novel therapeutic strategies.

In summary, this study identified PyTRAg7 as a key protein that induces host immune responses during malaria infection. PyTRAg7 binds to the macrophage surface receptor CD71 and modulates proinflammatory signaling by activating the NF-κB pathways, increasing parasitemia, and reducing host survival *in vivo*. These findings elucidate the molecular mechanisms by which PyTRAg7 promote host inflammatory responses and aggravate the malaria pathogenesis. More importantly, our work moves beyond the existing model focusing on erythrocyte invasion and cytoadherence, expand the understanding of interactions between *Plasmodium* and macrophages to advance prevailing models of parasite-host immune modulation.

### Limitations of the study

This study has several limitations. First, our study only focused on the rodent malaria parasite Py, to investigate the function of TRAg7 using a genetic deletion strategy. The roles of homologous TRAg7 proteins in Pv remain unexplored and warrant further investigation, which would deepen our understanding of host-Pv interaction. Secondly, although our results demonstrated that the absence of TRAg7 decreased the production of proinflammatory cytokines and improved host survival, whether the reduction of the inflammatory response contributed to the alleviation of immunopathological damage needs to be further clarified in murine malaria model. Third, confirming the role of macrophages CD71 in the effect of TRAg7 deficiency on the infection outcome of malaria will further verify the critical implications of TRAg7 for malaria pathogenesis. In addition, although we demonstrated that TRAg7 activates the NF-κB p65 pathway, the precise molecular mechanisms underlying this activation require further elucidation. Future studies should determine whether PyTRAg7 enhances the interaction between CD71 and the IKK complex, thereby facilitating inflammatory responses in macrophages.

## Resource availability

### Lead contact

Further information and request regarding the resources should be directed to and will be fulfilled by the lead contact, Yang Cheng (woerseng@126.com).

### Materials availability

All unique reagents generated in this study are available upon reasonable request from the lead contact.

### Data and code availability

Mass spectrometry analysis raw data with search results have been deposited at Mendley Data and are publicly available as of the date of publication. The DOI (https://doi.org/10.17632/x6b4z58cfc.1) is listed in the key resources table. Any additional information required to reanalyze the data reported in this study is available from the lead contact upon request.

This study does not report original code.

## Acknowledgments

We thank FIGDRAW (www.figdraw.com) for the drawing of Graphical abstract.

This work was supported by 10.13039/501100001809National Natural Science Foundation of China (nos. 82560401 and 82502749), 10.13039/501100002858China Postdoctoral Science Foundation Funded Project (no. 2023M731343), Scientific Research Program of Jiangsu Province Health Commission (no. x202321), Top Talent Support Program for young and middle-aged people of Wuxi Health Committee (no. HB2023039), the 10.13039/100000865Bill & Melinda Gates Foundation Malaria R&D sourcing & facilitation in China (no. INV-061480), the open topic of National Health Commission Key Laboratory of Parasite and Vector Biology (no. NHCKFKT2024-6), the 10.13039/501100004608Natural Science Foundation of Jiangsu Province (no. BK20231494), Wuxi Medical Key Discipline (no. ZDXK2021002), and Clinical Medical Research Project of the Regional Medical Center of Jiangnan University Affiliated Hospital and Donghai People’s Hospital.

## Author contributions

Y.S., Z.C., C.D., and Y.L. designed the experiments, analyzed the data, and wrote the manuscript supported by Y.C. and S.H.; C.D., Y.L., and Z.C. performed most experiments. Y.S., H.Z., and J.L. supported the knockout experiments. Y.S., B.W., S.Z., and X.H. prepared the figures. Y.C., Z.L., and J.C. provided suggestions for writing.

## Declaration of interests

The authors declare that they have no conflict of interest.

## STAR★Methods

### Key resources table

**Table:** 

REAGENT or RESOURCE	SOURCE	IDENTIFIER
**Antibodies**		

Goat Anti-Mouse IgG-HRP	SouthernBiotech	Cat#1030-05; RRID: AB_2619742
Goat Anti-Rabbit IgG-HRP	SouthernBiotech	Cat#4030-05; RRID: AB_2687483
Mouse anti-His antibody	Abclonal	Cat#ab18184; RRID: AB_2819175
Rabbit anti-GFP antibody	Cell Signaling Technology	Cat#2956T; RRID: AB_1196615
Rabbit anti-Flag antibody	Proteintech	Cat#20543-1-AP; RRID: AB_2572314
Rabbit anti-p65 antibody	Cell Signaling Technology	Cat#8242T; RRID: AB_10859369
Rabbit anti-p-p65 antibody	Cell Signaling Technology	Cat#3033T; RRID: AB_330559
Rabbit anti-p38 antibody	Cell Signaling Technology	Cat#8690T; RRID: AB_10999090
Rabbit anti-p-p38 antibody	Cell Signaling Technology	Cat#4511T; RRID: AB_2139682
Rabbit anti-ERK antibody	Cell Signaling Technology	Cat#4695T; RRID: AB_390779
Rabbit anti-ERK antibody	Cell Signaling Technology	Cat#4370T
Rabbit anti-JNK antibody	Cell Signaling Technology	Cat#9252T; RRID: AB_2250373
Rabbit anti-p-JNK antibody	Cell Signaling Technology	Cat#4668T; RRID: AB_823588
Rabbit anti-p-IKKα/β antibody	Cell Signaling Technology	Cat#2697T; RRID: AB_331626
Rabbit anti-GAPDH antibody	Zenbio	Cat#R380626; RRID:AB_369773
Anti-Mouse CD45 BG Violet 450 antibody	BioGems	Cat#07512-40; RRID: AB_2812239
Anti-Mouse CD3e PE antibody	BioGems	Cat#145-2C11; RRID: AB_2812224
Anti-Mouse F4/80 Antigen FITC antibody	BioGems	Cat#02922-50; RRID: AB_2812234
APC-conjugated anti-His Tag Antibody	BioLegend	Cat#362605; RRID: AB_2563346
Anti-Mouse PE/Cyanine CD19 antibody	BioGems	Cat#11212-77; RRID: AB_2812231
Goat anti-rabbit 488 fluorescent antibody	Invitrogen	Cat#A11008; RRID: AB_143165
Donkey anti-rabbit 568 fluorescent antibody	Invitrogen	Cat#A10042; RRID: AB_2534017
Anti-Human/Mouse CD11b Purified antibody	BioGems	Cat#03221-20; RRID: AB_2812228
CD71 (Transferrin Receptor) Monoclonal Antibody	Invitrogen	Cat#14-0711-82; RRID: AB_467319

**Bacterial and virus strains**		

*Escherichia coli* DH5α	TransGen Biotech	Cat#CB101-01
*Escherichia coli* BL21 (DE3)	TransGen Biotech	Cat#CB106-02

**Chemicals, peptides, and recombinant proteins**		

isopropyl β-*d*-1-thiogalactopyranoside (IPTG)	TransGen Biotech	Cat#GF101-01
CCK8	Beyotime	Cat#C0038
Fluorescence quenching tablet containing DAPI	Vector Laboratories	Cat#SP-8500-15
Hieff® qPCR SYBR Green Master Mix (No Rox)	YEASEN	Cat#11201ES08
Western and IP lysis buffer	Beyotime	Cat#P0013
Pyrimethamine	MACKLIN	Cat#58-14-0
4% paraformaldehyde	Meilunbio	Cat#MA0192-1
Hieff Trans® Liposomal 2000 Transfection Reagent	YEASEN	Cat#40802ES03
Tween 20	Solarbio	Cat#T8220
Trans2K® Plus DNA Marker	TransGen Biotech	Cat#BM111-01
180 kDa Prestained Protein Marker	Vazyme	Cat#MP102-01
Tris	BioFroxx	Cat#1328GR100
Sodium dodecyl sulfate(SDS)	Solarbio	Cat#S8010
Glycine	MeilunBio	Cat#mb4166
Ni-NTA Agarose	QIAGEN	Cat#30210
Acetone	Sinopharm Group Chemical Reagent Co., Ltd.	Cat#10000418
TEV Protease (His-tag)	Beyotime	Cat#P2308
Percoll	Solarbio	Cat#P8370

**Critical commercial assays**		

ToxinEraser endotoxin removal kit	Genscript	Cat#L00338
ToxinSensor^TM^ Chromogenic LAL Endotoxin Assay Kit	Genscript	Cat#L00350
Rapid total RNA extraction kit	ES Science	Cat#RN001-50Rxns
Hifair® III 1st Strand cDNA Synthesis Kit	YEASEN	Cat#11139ES60
Nitric Oxide Assay Kit	Beyotime	Cat#S0021S
IL-1β ELISA assay kit	Elabscience	Cat#E-EL-M0037
IL-6 ELISA assay kit	Elabscience	Cat#E-EL-M0044
TNF-α ELISA assay kit	Elabscience	Cat#E-EL-M3063
Giemsa stain Kit	Nanjing Jiancheng Bioengineering Institute	Cat#D011-2-3
TransStart® FastPfu DNA Polymerase Kit	TransGen Biotech	Cat#AP221-01
PAGE Gel Quick Preparation Kit (10%)	YEASEN	Cat#20325ES62
ECL Ultra Kit	NCM Biotech	Cat# P10300
Fast Silver Stain Kit	Beyotime	Cat# P0017S
Universal Genomic DNA Kit	CWBIO	Cat#CW2298M

**Deposited data**		

Mass Spectrometry Analysis of raw data with Search Results list	This study	Mendeley Data: https://doi.org/10.17632/x6b4z58cfc.1

**Experimental models: Cell lines**		

Mouse: RAW264.7	National Collection of Authenticated Cell Cultures	CSTR:19375.09.3101MOUSCSP5036
Human: HEK293T	National Collection of Authenticated Cell Cultures	CSTR:19375.09.3101HUMGNHu17

**Experimental models: Organisms/strains**		

Mouse: BALB/c	Cavens	N/A
Mouse: ICR	Cavens	N/A
*Plasmodium yoelii* 17XL	Li Jian et al., 2011^45^	N/A
*Plasmodium yoelii* 17XL ΔTRAg7	This study	

**Oligonucleotides**		

Primers for plasmids constructs and parasite genotyping, see Table S1	This paper	N/A

**Software and algorithms**		

PlasmoDB	https://plasmodb.org/plasmo/	
Single-guide RNA designed website	http://zifit.partners.org/ZiFiT/CSquare9Nuclease.aspx	
Signal peptide (SP) predicted website	https://services.healthtech.dtu.dk/services/SignalP-5.0/	
Transmembrane domain (TM) predicted website	https://services.healthtech.dtu.dk/services/TMHMM-2.0/	
FlowJo	BD Biosciences	RRID:SCR_008520
ImageJ	NIH	RRID:SCR_003070

### Experimental model and participant details

#### Experimental animals, parasites and cell lines

BALB/c and ICR mice (Cavens Laboratory Animal) were used in this study. All experiments were conducted using 6-week-old female mice (*n* = 8 per group). All of the mice were housed in pathogen-free conditions, and handled in accordance with the requirements of the Guideline for Animal Experiments. The mice experiments used in accordance with guidelines approved by Animal Ethics Committee of Jiangnan University (JN.No.20200530t1200930[083]).

*Trag7* knockout parasites were derived from the Py17XL strain. Experimental mice were infected intraperitoneally with 1 × 10^6^ Py 17XL–parasitized RBCs. Following infection, the mice were monitored daily to assess their survival. Tails' blood was collected to prepare thin blood smears, which were stained with Giemsa and microscopically examined to determine parasitemia.

The mouse monocyte/macrophage cell line RAW264.7 and the human embryonic kidney epithelial cell line HEK293T were obtained from the Cell Bank of the Chinese Academy of Sciences (Shanghai, China). Cells were not authenticated or tested for mycoplasma contamination. RAW264.7 macrophages and HEK293T cells were seeded in culture plates under standard culture conditions (37°C, 5% CO_2_).

### Method details

#### Expression and purification of recombinant PyTRAgs

The nucleotide sequences encoding PyTRAg were obtained from the PlasmoDB website (https://plasmodb.org/plasmo/; PyTARg1: PYYM_0625300; PyTARg2: PYYM_0625000; PyTARg7: PYYM_0701200; PyTARg8: PYYM_0524800; PyTARg11: PYYM_0524900). To achieve an efficient recombinant expression and purification of PyTRAgs, we constructed the recombinant protein fragments without the predicted signal peptide (SP) (predicted using https://services.healthtech.dtu.dk/services/SignalP-5.0/) and the transmembrane domain (TM) (predicted using https://services.healthtech.dtu.dk/services/TMHMM-2.0/), as Table S1. Therefore, the interaction partners identified in this study should be interpreted as interactions with specific segments of the PyTRAgs proteins. We acknowledge that this approach may not capture interactions that rely on TMs or the complete membrane environment.

They were synthesized by TianLin Biotech (Wuxi) with codon optimization for expression in the *Escherichia coli* DH5α (TianGen Biotech) and cloned into the pET30a vector (primer sequences in Table S2).

Recombinant plasmids of PyTRAgs were transformed into *E*. *coli* BL21 (DE3) cells (TransGen Biotech) and grown in Luria–Bertani broth at 37°C. The culture was induced with 0.1 mM isopropyl β-*d*-1-thiogalactopyranoside (IPTG) (TransGen Biotech) at 600 nm (OD600), reached 0.6, and allowed to grow for another 8 h at 37°C. The bacterial pellet was collected via centrifugation and lysed by ultrasonication, followed by the purification of His-PyTRAg protein using Ni-NTA column chromatography (Wuxi TianLin Biotech). Purified recombinant proteins were analyzed using 10% sodium dodecyl sulfate-polyacrylamide gel electrophoresis (SDS-PAGE) and western blotting analysis using an anti-His (Cell Signaling Technology) monoclonal antibody. Endotoxin in the purified protein was removed by using a ToxinEraser endotoxin removal kit (Genscript). Then, the endotoxin concentration of recombinant proteins was measured using the ToxinSensor Chromogenic LAL Endotoxin Assay Kit (Genscript). The endotoxin levels in the recombinant proteins TRAg1, TRAg2, TRAg7, TRAg8, and TRAg11 were 0.001533, 0.0014506, 0.0015937, 0.0014149, and 0.001379 EU/mL, respectively. The purity of the PyTRAgs (PyTRAg1, PyTRAg2, PyTRAg7, PyTRAg8, and PyTRAg11) recombinant proteins obtained was 90, 80, 85, 95, and 80%, respectively. The concentrations of the purified PyTRAgs protein were determined to be 2, 1.4, 1, 3, and 1.6 mg/mL, respectively.

#### Preparation of polyclonal antibodies

The preparation of polyclonal rabbit antibodies involved emulsifying purified PyTRAgs protein with an equal volume of adjuvant, followed by administration via multiple intramuscular injections into the thigh muscles of New Zealand White rabbits. A series of three immunizations was conducted at 28-day intervals, with the initial immunization using complete Freund’s adjuvant and the subsequent immunizations using incomplete Freund’s adjuvant. After 2 weeks of the final immunization, rabbit blood samples were collected from the eyes and centrifuged at 2000 rpm for 10 min, and the supernatant was stored at −80°C.

Rabbit IgG from each serum sample was purified by Shanghai YouLong Biological Co., Ltd. Then, the lysate of the infected red blood cells was considered as the sample, and western blotting was used to detect the specificity of the polyclonal antibodies to recognize the corresponding natural PyTRAg proteins.

#### SDS-PAGE and western blotting

Purified recombinant proteins were mixed with 1× SDS-reducing loading buffer, boiled for 10 min at 100°C, and resolved on 10% SDS-PAGE to perform Coomassie brilliant blue staining. For western blotting analysis, the proteins were sampled at 20 μg, separated by SDS-PAGE, and transferred onto polyvinylidene difluoride membranes. The membranes were blocked with 5% skimmed milk for 2 h at room temperature (RT). The membranes were incubated with primary antibodies overnight at 4°C and horseradish peroxidase-labeled secondary antibody (1:5000, SouthernBiotech) for 1.5 h at RT. After washing the membrane thrice with Tris-buffered saline containing 0.05% Tween 20 (TBST), the enhanced chemiluminescence (ECL) Ultra Kit (NCM Biotech) was used to visualize the bands by ChemiDoc (BioRad). Mouse anti-His antibody was sourced from ABclonal; and rabbit anti-Flag antibodies were procured from Proteintech; anti-p65, anti-p-p65, anti-p38, anti-p-p38, anti-extracellular signal-regulated kinase (ERK), anti-p-ERK, anti-c-Jun N-terminal kinase (JNK), anti-*p*-JNK, and rabbit anti-*p*-IKKα/β antibodies rabbit anti-GFP antibody were obtained from Cell Signaling Technology.; Rabbit anti-CD71 antibody was obtained from Thermo Fisher Scientific and Rabbit IgG isotype antibody (Abcam) was used as an isotype control. The protein expression was semi-quantified using the ImageJ software.

RAW264.7 cells (2 × 10^5^ cells/well) were seeded in 12-well plates and cultured for 12 h. Cells were treated with different concentrations of PyTRAg7 (1, 5, 10, 20, and 50 μg/mL) for 48 h. Subsequently, the cells were lysed for Western blotting analysis. The protein expression was quantified using the ImageJ software. To verify whether *trag7* gene was knocked out, the polyclonal antibodies prepared by immunizing rabbits with the recombinant PyTRAg7 and PyMSP8 proteins were respectively used to detect the native proteins in the lysates of WT and ΔTRAg7 strains-infected RBCs.

#### Flow cytometry

To detect the binding of PyTRAg protein to the spleen cells, 2 × 10^6^ splenocytes were removed from each EP tube, and control (spleen cells) and experimental groups (spleen cells +40 μg/mL PyTRAg1, PyTRAg2, PyTRAg7, PyTRAg8, PyTRAg11 recombinant proteins) were set up with three replicates. Considering that the PyTRAg protein is expressed as a His-tag fusion, the cells were stained with allophycocyanin (APC)-conjugated anti-His Tag Antibody (BioLegend; diluted 1:200 in PBS) on an ice bath and protected from light for 15 min. After staining, the cells were washed thrice with PBS and centrifuged at 400 *g* for 10 min at 4°C to remove the unbound antibody. Flow cytometry data were analyzed using FlowJo software. The fluorescence intensity of the bound protein was then measured by flow cytometry (BD FACSAria III).

To detect the binding of PyTRAg proteins to the macrophage, 2 × 10^6^ RAW264.7 cells were taken from each EP tube, and control (RAW264.7 cells) and experimental groups (RAW264.7 cells +40 μg/mL Vector, PyTRAg1, PyTRAg2, PyTRAg7, PyTRAg8, PyTRAg11 recombinant proteins) were set up with three replicates. The pET30a vector without the insert fragment served as the negative control, which could express a histidine-tagged protein in the prokaryotic expression system. Flow cytometry data were analyzed as described above.

To further determine which cell type was mainly bound by the recombinant TRAg protein in primary spleen cells, mouse spleen immune cells were extracted. Mice were anesthetized and killed and sterilized by soaking in 75% alcohol for 5 min. Spleen tissues were taken out and put into a Petri dish containing 3 mL of PBS, and a filter was placed on top of the Petri dish and underneath the spleen tissues. Mice spleens were fully ground by a grinding rod, and the cell suspension was collected into a centrifuge tube and centrifuged at 300 *g* for 5 min at RT, the supernatants were discarded. 3 mL of erythrocyte lysis solution was added to the collected cells. And the lysate was blown gently to mix, lysed at RT for 5 min, quickly added 10 mL of PBS to terminate lysis, centrifuged at 300 *g* for 5 min, discarded the supernatant, resuspended the cells in complete medium, counted and retained. Then the cells were incubated with or without recombinant protein TRAg7 in a mixer at RT for 2 h TRAg1 protein as the negative control. Since TRAg7 and TRAg1 proteins labeled with His-tag, we used APC-anti-His antibody to detect the cells interacting with TRAgs after co-incubation. Dilute Anti-Mouse CD45 PerCP-Cy5 antibodies, Anti-Mouse CD3e PE antibodies, Anti-Mouse CD19 PE/Cyanine antibodies, Anti-Mouse F4/80 Antigen FITC antibodies, Anti-Mouse CD11 b PE antibodies and APC-His antibodies (BioGems) APC with PBS at 1:200, respectively. Then the cells were incubated on ice for 15 min from light, washed with pre-cooled PBS for three times, and centrifuged at 400 g at 4°C for 10 min. And the binding of TRAg7 to T cells, B cells and macrophages was analyzed by flow cytometry (BD FACSAria III). Flow cytometry data were analyzed as above.

#### Cell counting kit-8 (CCK8) assay

RAW264.7 cells (1 × 10^3^ cells/well) were seeded into a 96-well plate and cultured for 12 h. The cells were treated with lipopolysaccharide (1 μg/mL) and different concentrations of PyTRAg7 (1, 5, 10, and 50 μg/mL) for 48 h. Cell proliferation was measured using the CCK8 (Beyotime) and incubated for 3 h. The optical density of each well was measured by using a microplate spectrophotometer (BioTek) at 450 nm. Four independent experiments were conducted.

#### Immunofluorescence assays

For the p65 nuclear-translocation experiment, the primary antibody used was an anti-p65 rabbit antibody (1:200, Cell Signaling Technology), and the secondary antibody was a goat anti-rabbit 488 fluorescent antibody (1:400, Invitrogen). The cell sample was fixed with pre-cooled acetone (Sinopharm Group Chemical Reagent Co., Ltd.) for 15 min at RT and blocked with 5% skimmed milk for 1 h. The cells were subsequently probed with primary antibodies in a wet box, incubated at 37°C for 1 h, and washed thrice with 4°C pre-cooled PBS containing 0.05% Tween 20 (PBST). Goat anti-rabbit 488 fluorescent antibody (1:400, Invitrogen) was used as the secondary antibody. It was incubated in a wet-box dark chamber at 37°C for 30 min and washed thrice with PBS. An anti-fluorescence quenching tablet containing 4,6-diamidino-2-phenylindole (DAPI) (Vector Labs) was added to each well and imaged using confocal microscopy (Zeiss). The following primary antibodies were used: anti-PyTRAgs antibody (1:100, YouLong Biotech) and anti-p65 antibody (1:200, Cell Signaling Technology).

To determine whether TRAg7 localizes on the RBC surface, the samples of Py-infected RBCs were prepared. The cell sample was fixed with pre-cooled 4% paraformaldehyde fixative for 15 min at RT to keep the cell membranes intact and blocked with 5% skimmed milk for 1 h. The primary antibodies were anti-PyTRAg7 mouse antibodies and anti-Band3-Loop5 (extracellular region of Band3) rabbit antisera (1:100, YouLong Biotechnology). The secondary antibodies used were goat anti-mouse 488 fluorescent and donkey anti-rabbit 568 fluorescent antibodies (1:400; Invitrogen). The samples were probed with primary antibodies in a wet box, incubated at 37°C for 1 h, and washed thrice with pre-cooled PBST at 4°C. Then, the samples were incubated with secondary antibodies for immunofluorescence in a humidified dark chamber at 37°C for 30 min, followed by three washes with PBST. DAPI was added to each well and imaged using a confocal microscope (Zeiss).

#### Quantitative real-time PCR (qRT-PCR)

A total of 1 × 10^5^ RAW264.7 cells were added to a 48-well plate and cultured for 12 h in an incubator. Furthermore, PyTRAg7 or PyTRAg2 recombinant protein (10 μg/mL) was added and stimulated for 48 h. RNA samples were extracted from RAW264.7 cells using a total RNA kit (ES Science). The resulting total RNA was used to obtain cDNA using Hifair III 1st Strand cDNA Synthesis Kit (YEASEN). Each sample was assayed in triplicate. RT-PCR was performed using 2× qPCR SYBR Green Master Mix (YEASEN) in a LightCycler480 II apparatus (Roche). The thermal cycling conditions were as follows: initial denaturation at 95°C for 30 s, followed by 40 cycles of PCR at 95°C for 5 s and 60°C for 30 s, then 95°C for 5 s and 60°C for 1 min, followed by 50°C for 30 s. Glyceraldehyde 3-phosphate dehydrogenase served as an internal control, and the ratio of each target gene was determined by Light cycle 480 PCR instrument (Roche). Data were analyzed using the ^△△^CT method. The primer sequences used in the study are listed in Table S1.

#### Enzyme-linked immunosorbent assay (ELISA)

A total of 1 × 10^5^ RAW264.7 cells were seeded into 48-well plates and cultured for 12 h in an incubator. Additionally, PyTRAg7 or PyTRAg2 recombinant protein (10 μg/mL) was added to the cultures and incubated for 48 h. Each sample was assayed in triplicate. Then, RAW264.7 macrophages were incubated for 48 h. Cell supernatants were collected in EP tubes for measurement of IL-1β, IL-6, and TNF-α using commercial enzyme-linked immunosorbent assay kits (Elabscience). Absorbance was measured at 450 nm using the BioTek microplate spectrophotometer.

#### Measurement of NO production

NO production was measured using Nitric Oxide Assay Kit (Beyotime). The procedures were performed strictly according to the kit instructions. Measure the absorbance at 540 nm using the BioTek microplate spectrophotometer. And calculate the concentration of NO in samples from the standard curve.

#### Plasmid construction

Primers were designed to amplify CD71, HSP60, and CKAP4 from cDNA synthesized from RAW264.7 cell mRNA using the Hifair III First Strand cDNA Synthesis Kit (YEASEN). Table S1 lists all primer sequences. PCR products were cloned into the XhoI and SalI restriction sites of the pEGFP-C1 vector. Plasmid sequencing (Wuxi TianLin Biotech) confirmed the absence of mutations.

#### Pull-down assay and LC-MS analysis

To screen candidate PyTRAg7-interacting proteins, recombinant PyTRAg7 was purified by Ni-NTA affinity chromatography and co-incubated with membrane proteins (400 μg, 2 mg/mL) extracted from RAW264.7 cells. After removal of nonspecifically bound proteins, TRAg7-binding partners were analyzed by SDS-PAGE and visualized using the Fast Silver Stain Kit (Beyotime). Specific protein bands were excised and submitted to Shanghai Applied Protein Technology Company for LC-MS analysis.

Then, excised gel bands were destained, dehydrated, reduced with 10 mM DTT (37°C, 60 min), and alkylated with 60 mM IAA (room temperature, dark, 60 min). Following serial washes and lyophilization, in-gel digestion was performed with trypsin (2.5–10 ng/μL) at 37°C for 20 h. Peptides were extracted using 60% (v/v) ACN containing 0.1% (v/v) TFA under sonication, combined with the initial supernatant, lyophilized, and reconstituted in 2% (v/v) FA. Peptides were separated on an EASY-nLC system (Thermo Scientific) equipped with a trap column (Acclaim PepMap100, C18) and an analytical column (EASY column, 15 cm,150 μm ID, 3 μm, C18). Separation was achieved at 300 nL/min using a 60-min gradient of solvent B (84% ACN, 0.1% FA): 0–60% for 50 min, 60–90% for 4 min, and 90% for 6 min. Eluted peptides were analyzed on a Q-Exactive mass spectrometer (Thermo Scientific) in positive ion mode. Full MS scans (m/z 300–1800) were acquired at a resolution of 70,000. The top 20 most intense precursor ions (AGC target 1 × 10^6^, dynamic exclusion 30 s) were selected for higher-energy collisional dissociation (HCD). MS/MS spectra were acquired at a resolution of 17,500. Raw data were analyzed using Proteome Discoverer Daemon 2.5 (Thermo Scientific).

Moreover, HEK293T cells were transfected with pEGFP-C1, pEGFP-CD71, pEGFP-CKAP4, or pEGFP-HSP60 using Hieff Trans Liposomal 2000 Transfection Reagent (YEASEN). Transfected cells were lysed with cell lysis buffer for western blotting and IP (Beyotime), supplemented with protease inhibitors. Lysates were incubated with PyTRAg7 protein for 12 h at 4°C and with constant rotation. Beads were washed three times with prechilled isotonic and hypertonic buffer to remove nonspecific interactions. Bound proteins were eluted using imidazole solutions at 100, 200, 300, 400, and 500 mM, and eluates were analyzed by western blotting. To remove the His-tag, purified recombinant PyTRAg7 was incubated with TEV Protease (His-tag) at 4°C for 12 h with constant rotation. The His-tagged protease and uncleaved proteins were then depleted using Ni-NTA resin. Following cleavage, the His-tagged TEV protease and any un-cleaved proteins were removed using Ni-NTA resin to obtain tag-free PyTRAg7. HEK293T cell lysates transfected with pEGFP-CD71 were incubated with either His-tagged or tag-free PyTRAg7 (200 µg/mL) for pull-down assays.

Fresh mouse erythrocyte membrane proteins were extracted.^46^ The binding of PyTRAg7 to natural CD71 protein was confirmed through the His-pull-down experiment. Erythrocyte membrane proteins were incubated as prey proteins together with PyTRAg1 or PyTRAg7 fixed on magnetic beads. After washing, perform western blotting as mentioned before.

#### Assay of neutralizing antibody effect

5 × 10^5^ RAW264.7 cells were taken and incubated at room temperature with anti-CD71 antibodies of different concentrations (diluted by 1:100, 1:50 and 1:20) for 2 h to block the CD71 receptor on the surface of macrophages. Then, PyTRAg7 recombinant protein was added at room temperature for 2 h. After lysis of the cells, western blotting analysis was performed.

#### Malaria parasite gene knockout

The *Pytrag7* sequence was obtained from the PlasmoDB database (https://plasmodb.org/plasmo/). To construct the *pytrag7* deletion plasmid, 500 consecutive bases from the 5′ and 3′ untranslated regions flanking the *pytrag7* open reading frame were selected for amplification of the homology arms (primer sequences are listed in Table S1). The left and right homology arms were ligated into the plasmid by In-Fusion cloning. Single-guide RNA sequences targeting *pytrag7* were designed using the website (http://zifit.partners.org/ZiFiT/CSquare9Nuclease.aspx), protective bases were added, and the single-guide RNA fragments were inserted into the plasmid by blunt-end ligation.^47^ A schematic diagram of the knockout plasmid is shown in Figure S4A. Purified schizont-stage parasites (5 μL) and 5 μg of the knockout plasmid were mixed in sterile EP tubes, and electroporation buffer was added to a final volume of 100 μL. Transfection was performed using the Lonza 2b electroporator (Lonza BioResearch) with the T-16 program. Electroporation products containing the transfected parasites were injected into ICR mice (Cavens Laboratory Animal) via the tail vein in 200 μL of PBS. After 24–36 h, tail blood smears were prepared and stained with Giemsa stain kit (Nanjing Jiancheng Bioengineering Institute). When parasites were detected microscopically, pyrimethamine (6 μL/mL; MACKLIN) was added to select for positive clones. Gene deletion was verified by western blotting, PCR, and DNA sequencing. Table S1 lists all primer sequences. Genomic DNA was extracted using the Universal Genomic DNA Kit (CWBIO). PCR amplification was performed using TransStart FastPfu DNA Polymerase (TransGen Biotech) under the following conditions: initial denaturation at 95°C for 30 s; 35 cycles of 95°C for 10 s, 60°C for 10 s, and 72°C for 30 s; and a final extension at 72°C for 5 min. PCR products were analyzed by agarose gel electrophoresis. For western blotting, anti-PyTRAg7 and anti-PyMSP8 antisera were generated using standard procedures and applied as primary antibodies. Since PyMSP8 is a GPI-anchored membrane protein of Py trophozoites and merozoites,^48^ it was used to be the “reference protein” for knockdown verification. DNA samples were submitted to TianLin Biotech for sequencing to confirm targeted deletion of the *PyTRAg7* coding region.

#### Parasites growth *in vitro*

Upon the parasitemia level in infected mice surpassing 70%, predominantly during the schizont stage, blood was extracted from the ocular vein utilizing an anticoagulant. RBCs were subsequently isolated through centrifugation, washed, and resuspended in RPMI 1640 medium. The RBC suspension was then subjected to filtration through an NWF filter at a controlled flow rate to eliminate leukocytes, followed by rinsing to recover the remaining RBCs. Post-centrifugation, the RBC pellet was layered onto 72% Percoll and centrifuged under specified conditions (400*g*, 30 min, 20°C, with both acceleration and deceleration set to zero). The intermediate layer, enriched with schizonts, was collected and washed. The schizonts were enriched from the blood of infected mice by Percoll density centrifugation and incubated with normal mouse RBCs at 37°C and 5% CO_2_ for 6, 12, 18, and 24 h. Invasion into new RBCs was quantified on Giemsa-stained thin smears by scoring ring-stage parasites.^49^

#### Hematoxylin and eosin staining

Mouse spleens were fixed in 4% paraformaldehyde (Meilunbio) for 24 h. Fixed tissues were placed in embedding cassettes and rinsed under running water for 1 h. Samples were dehydrated using an automated tissue processor with the following program: 75% ethanol for 1 h, 80% ethanol for 1 h, 95% ethanol for 1 h, absolute ethanol I for 30 min, absolute ethanol II for 30 min, xylene I for 15 min, and xylene II for 15 min. The embedding frame was removed and placed in a wax cylinder, where tissues were immersed in molten paraffin for 45 min in wax baths A and B. The solidified tissue blocks were mounted on a microtome, sectioned at 5 μm thickness, and baked at 55°C for 30 min, followed by an additional 1 h in a 55°C oven. The sections were deparaffinized in xylene for 8 min and rehydrated through a graded ethanol series (absolute ethanol I and II, 95% ethanol, 85% ethanol, and 75% ethanol; 3 min each), then rinsed in running water for 5 min. After hematoxylin and eosin staining, slides were rinsed in running water, counterstained with eosin for 1 min, and rinsed again. Samples were dehydrated through graded ethanol solutions (100%, 95%, 75%, and 50%), cleared in xylene, mounted with neutral resin, and examined under the microscope (Nikon).

### Quantification and statistical analysis

All experimental data are presented as mean ± SEM and were analyzed using GraphPad Prism version 5. One-way analysis of variance (ANOVA) was used for comparisons among multiple groups. The Tukey’s multiple comparisons test was performed for pairwise comparisons. A Student’s *t* test was used to compare two independent groups. Statistical significance was defined as ∗*p* < 0.05, ∗∗*p* < 0.01, ∗∗∗*p* < 0.001, and ∗∗∗∗*p* < 0.0001; ns indicates no significant difference.
