# Supplementary material for: Interaction of Plasmodium yoelii tryptophan-rich antigen 7 with CD71 on macrophage membrane regulates host inflammatory response
Source: iScience. 2026 Mar 25;29(4):115468. doi: 10.1016/j.isci.2026.115468 (PMC13091410; doi:10.1016/j.isci.2026.115468)
Supplement: Document S1. Figures S1–S5, Tables S1, and S2 [file mmc1.pdf]

iScience, Volume *29*

Supplemental information

Interaction of *Plasmodium yoelii* tryptophan-rich antigen 7 with CD71 on macrophage membrane regulates host inflammatory response

Yifan Sun, Zhe Chen, Chenyan Du, Yao Lei, Jian Li, Hangye Zhang, Xuan Huang, Bo Wang, Shenghuan Zuo, Zhiyue Lv, Jianping Cao, Su Han, and Yang Cheng

Supplemental Files

A

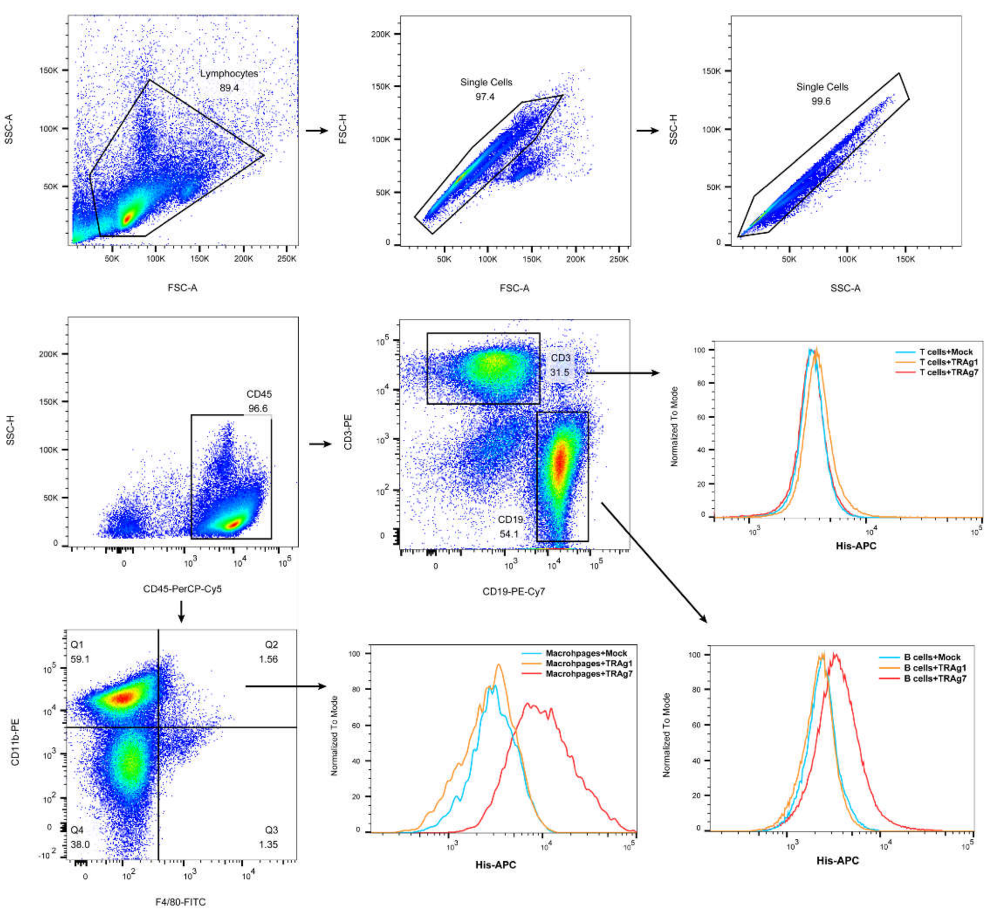

Figure S1. The binding ability of recombinant protein PyTRAg7 to primary macrophages, T cells and B cells. (Related to Figure 1)

Gating strategy to identify macrophages, B cell and T cells in the spleen and detect the binding ability of recombinant protein PyTRAg7 and PyTRAg1 to primary cells using flow cytometry.

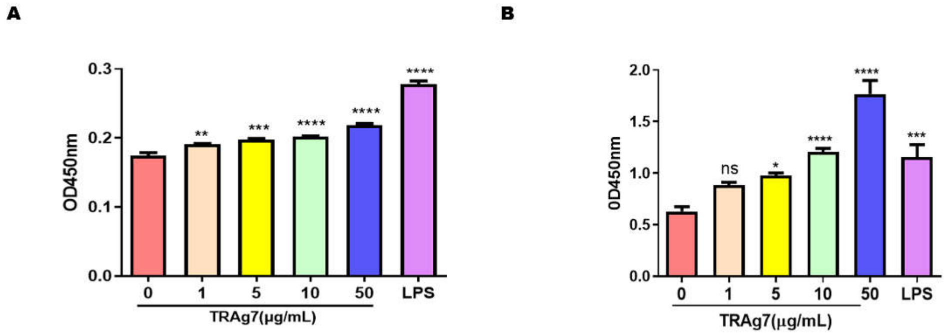

Figure S2. Effects of PyTRAg7 on the proliferation of spleen cells and macrophages. (Related to Figure 1)

(A) The spleen cells were added to 96-well plates and stimulated with PyTRAg7 recombinant protein (0, 1, 5, 10, and 50 μg/mL) for 48 h. The spleen cell proliferation was measured using CCK8 assay.

(B) RAW264.7 cells were treated with PyTRAg7 protein (0, 1, 5, 10, and 50 μg/mL) for 48 h, and cell proliferation was measured using CCK8 assay.

Data are represented as mean ± SEM. One-way ANOVA was used to compare multiple groups of samples. (ns, no significance; *$P < 0.05$, **$P < 0.01$, ***$P < 0.001$, ****$P < 0.0001$).

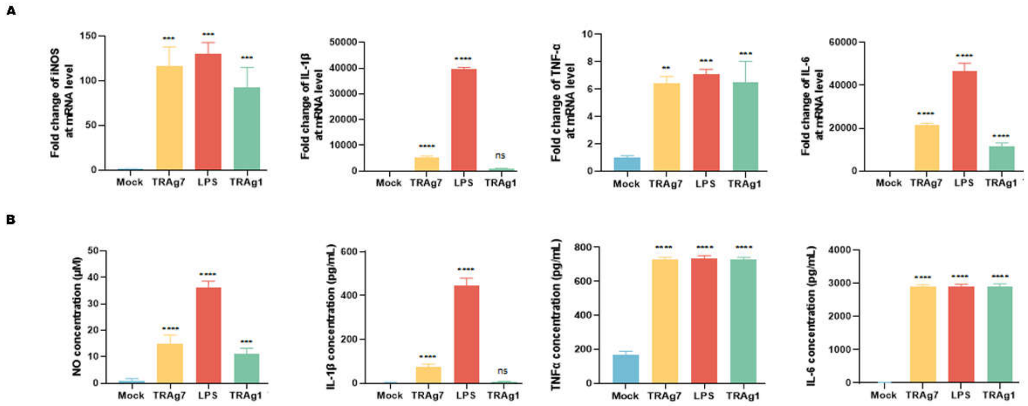

Figure S3. TRAg7 induces the expression of proinflammatory cytokines in macrophages. (Related to Figure 2)

(A) The mRNA level of iNOS, IL-1β, IL-6, and TNF-α in RAW264.7 cells after PyTRAg7 and PyTRAg1 recombinant protein (10 μg/mL) stimulation for 48 h, as detected by qRT-PCR. GAPDH was used as an internal control. LPS was used as the positive control.

(B) The protein level of IL-1β, IL-6, and TNF-α and the level of NO produced by RAW264.7 cells after PyTRAg7 and PyTRAg1 recombinant protein (10 μg/mL) stimulation for 48 h, as detected by ELISA. LPS was used as a positive control.

Data are represented as mean ± SEM. One-way ANOVA was used to compare multiple groups of samples. (ns, no significance; $^{**}P < 0.01$, $^{***}P < 0.001$, $^{****}P < 0.0001$).

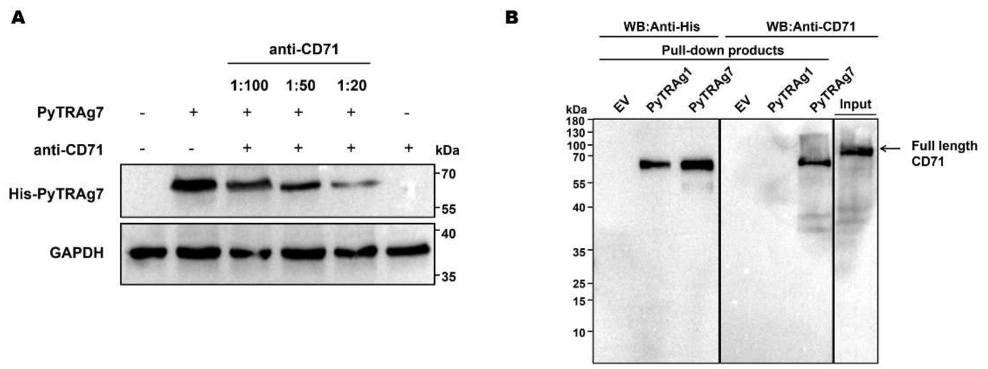

Figure S4. CD71 mediates the binding of PyTRAg7 to macrophages. (Related to Figure 3)

(A) The CD71 antibody attenuated the binding of PyTRAg7 to macrophages.

(B) Interactions of PyTRAg7 to native CD71 confirmed by His-pull-down assays.

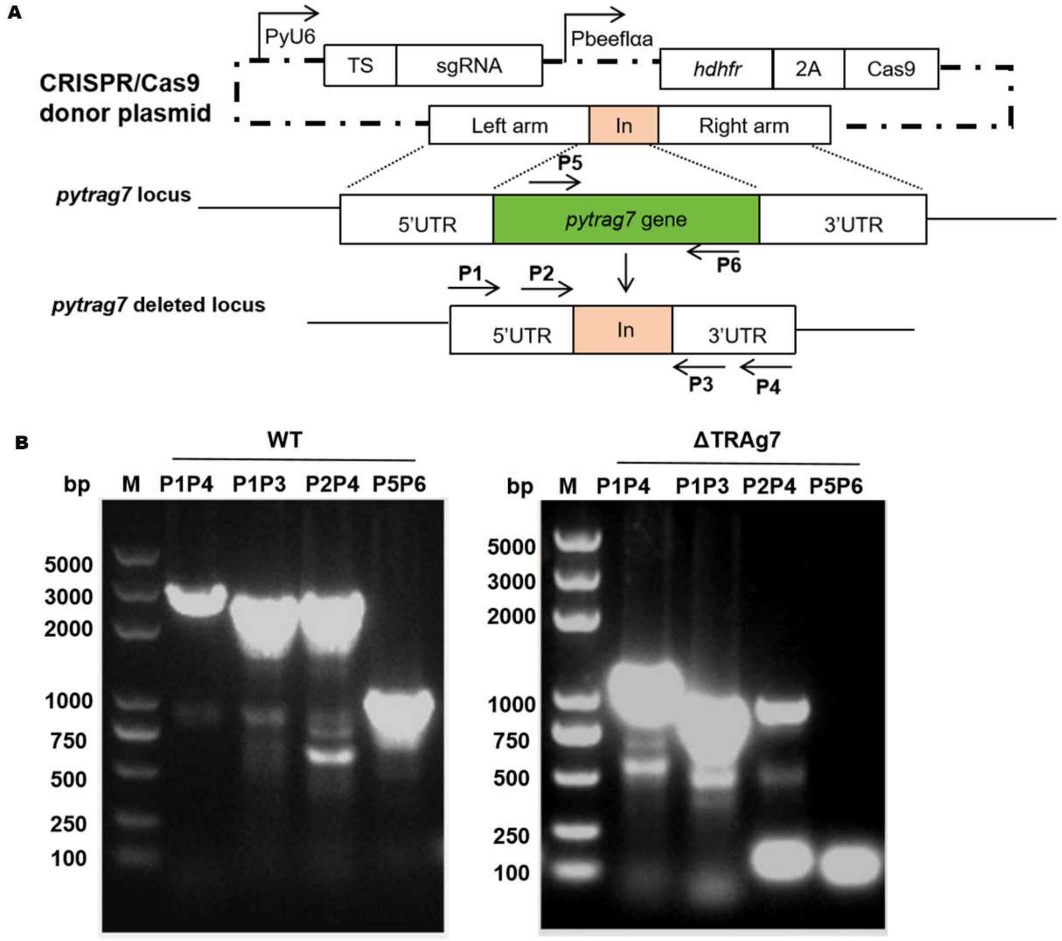

Figure S5. Schematic diagram showing the strategy to construct PyTRAg7 knockout stain and PCR identification. (Related to Figure 4)

(A) Schematic construct for disrupting the *Pytrag7* gene.

(B) PCR analysis of in WT and ΔTRAg7 strain of Py17XL parasites. The positions of primers (e.g., P1/P4) are shown in panel A.

Table S1. Recombinant protein fragments of PyTRAgs family used in this study.

Genes	Full-length (aa)	Predicted SP (aa)	Predicted TM (aa)	Expressed fragment (aa)
PyTRAg1	373	1-28	7-29	30-373
PyTRAg2	549	-	36-58	59-549
PyTRAg7	424	-	-	1-424
PyTRAg8	354	-	-	1-354
PyTRAg11	341	-	40-50	51-341

Table S2. Amplified primers used in this study.

Primers	Sequences
pET30a-PyTRAg1-F	5'-**GCTGATATCGGATCC**ATGGCATCCT TCGATCCGTCTTCAG-3'
pET30a- PyTRAg1-R	5'-**GTGGTGGTGCTCGAG**CTTATCGTCG TCATCCTTGTAATC-3'
pET30a- PyTRAg2-F	5'-**GCTGATATCGGATCC**AATAAAAAAA CAAGAACGGTGATA-3'
pET30a- PyTRAg2-R	5'-**GTGGTGGTGCTCGAG**CTTATCGTCGT CATCCTTGTAAT-3'
pET30a- PyTRAg7- F	5'-**GCTGATATCGGATCC**AATGTAAAG CACCGTTTGAAGAC-3'
pET30a- PyTRAg7-R	5'-**GTGGTGGTGCTCGAG**CTTATCGTCG TCATCCTTGTAAT-3'
pET30a- PyTRAg8- F	5'-**GCTGATATCGGATCC**ATGTTCCAA CTGAACCCGTTTGACT-3'
pET30a- PyTRAg8-R	5'-**GTGGTGGTGCTCGAG**CTTATCGTC GTCATCCTTGTAATCT-3'
pET30a-PyTRAg11-F	5'-**GCTGATATCGGATCC**CTTATCGT CGTCATCCTTGTAAT-3'
pET30a-PyTRAg11-R	5'-**GTGGTGGTGCTCGAG**CTTATCGT CGTCATCCTTGTAAT-3'
pEGFP-CD71-F	5'- **GGACTCAGATCTCGAGCT** TGTAAGCGTGTAGAACAAAAAGA-3'
pEGFP-CD71-R	5'- **CGCGGTACCGTCGAC**AGCGTAATCTGGAAC ATCGTATGGGTAAAACTCATTGTCAATATTCCAAAT-3'
pEGFP-CKAP4-F	5'- **GGACTCAGATCTCGAGCT** CAGGTCCGGCGTGGC-3'
pEGFP-CKAP4-R	5'- **CGCGGTACCGTCGAC**AGCGTAATCTGGAAC ATCGTATGGGTA**GATCTTTTCATGGATCTTCTCAA**-3'
pEGFP-HSP60- F	5'- **GGACTCAGATCTCGAGCT** ATGCTTCGACTACCCACAGT-3'
pEGFP-HSP60-R	5'- **CGCGGTACCGTCGAC**AGCGTAATCTGGAAC ATCGTATGGGTAGAACATGCCGCCTCCC-3'
mGAPDH-F	GAGCCAAACGGGTCATCATCT
mGAPDH-R	GAGGGGCCATCCACAGTCTT
miNOS-F	GAGCTCGGGTTGAAGTGGTATG
miNOS-R	GAAACTATGGAGCACAGCCACAT
mIL-1β-F	GGTGTGTGACGTTCCCATTAGAC
mIL-1β-R	CATGGAGAATATCACTTGTTGGTTGA
mIL-6-F	ACAACCACGGCCTTCCCTACTT
mIL-6-R	CACGATTTCCCAGAGAACATGTG
mTNF-α-F	AAGCCTGTAGCCCACGTCGTA

PYC-TRAg7-L-Forword primer	GCCAAGCTTGGTACCCCAAAAAACTCCAAATAATCAA
PYC-TRAg7-L-Reverse primer	CCGCGGGGACCATGGCCTTCTCAATGTTACTTTAATTATAATG
PYC-TRAg7-R-Forword prime	CCGCGGCCGCTCGAG GTATCTACTTTTCTCACATGATGACA
PYC-TRAg7-R-Reverse primer	AAACTTAAGGAATTCGCAGCACAAAAATATGCAAG
pytrag7-sgRNA-F	TATTGATAATATTATAGAACAAAT
pytrag7-sgRNA-R	AAACATTTGTTCTATAATATTATC
ΔTRAg7-P1	CCAAAAAACTCCAAATAATCAA
ΔTRAg7-P2	GTAATATATTGGCGAAATAAATATAAT
ΔTRAg7-P3	GCAAATAGACTAGAGCATATATGC
ΔTRAg7-P4	GATGCATTCACATATATGTTTATATATT
ΔTRAg7-P5	GAAGATAGCGAAAATGAATATACTG
ΔTRAg7-P6	CGTAACTGAGGGTTTTTAACAA

Bold: The homologous arm sequence of the amplified vector. Blue: HA-tag.
